# The Functional Role of IgA in the IgM/IgA-Enriched Immunoglobulin Preparation Trimodulin

**DOI:** 10.3390/biomedicines9121828

**Published:** 2021-12-03

**Authors:** Fabian Bohländer, Sabrina Weißmüller, Dennis Riehl, Marcus Gutscher, Jörg Schüttrumpf, Stefanie Faust

**Affiliations:** 1 Department of Analytical Development and Validation, Biotest AG, Landsteinerstraße 5, 63303 Dreieich, Germany; Fabian.Bohlaender@biotest.com (F.B.); Dennis.Riehl@biotest.com (D.R.); Marcus.Gutscher@biotest.com (M.G.); 2 Department of Translational Research, Biotest AG, Landsteinerstraße 5, 63303 Dreieich, Germany; Sabrina.Weissmueller@biotest.com; 3 Corporate R&D, Biotest AG, Landsteinerstraße 5, 63303 Dreieich, Germany; Joerg.Schuettrumpf@biotest.com

**Keywords:** IgA, FcαRI, IVIG, trimodulin, ITAMi, Fc receptors, neutrophils, phagocytosis, cytokines

## Abstract

In comparison to human immunoglobulin (Ig) G, antibodies of IgA class are not well investigated. In line with this, the functional role of the IgA component in IgM/IgA-enriched immunoglobulin preparations is also largely unknown. In recent years, powerful anti-pathogenic and immunomodulatory properties of human serum IgA especially on neutrophil function were unraveled. Therefore, the aim of our work is to investigate functional aspects of the trimodulin IgA component, a new plasma-derived polyvalent immunoglobulin preparation containing ~56% IgG, ~23% IgM and ~21% IgA. The functional role of IgA was investigated by analyzing the interaction of IgA with FcαRI, comparing trimodulin with standard intravenous IgG (IVIG) preparation and investigating Fc receptor (FcR)-dependent functions by excluding IgM-mediated effects. Trimodulin demonstrated potent immunomodulatory, as well as anti-pathogenic effects in our neutrophil model (neutrophil-like HL-60 cells). The IgA component of trimodulin was shown to induce a strong FcαRI-dependent inhibitory immunoreceptor tyrosine-based activation motif (ITAMi) signaling, counteract lipopolysaccharide-induced inflammation and mediate phagocytosis of *Staphylococcus aureus*. The fine-tuned balance between immunomodulatory and anti-pathogenic effects of trimodulin were shown to be dose-dependent. Summarized, our data demonstrate the functional role of IgA in trimodulin, highlighting the importance of this immunoglobulin class in immunoglobulin therapy.

## 1. Introduction

In humans, immunoglobulin A (IgA) is produced in higher amounts than all other immunoglobulin isotypes together. IgA is the most abundant antibody on mucosa (~74%) and the second most abundant in serum (15%) [1,2,3,4]. A large heterogeneity of IgA is observed: besides the monomeric form with two subclasses (IgA1 and IgA2), dimers and higher-order multimers (mainly tetramers), secretory forms and different glycoforms are known [2,4,5,6]. The covalent bound J-chain is fundamental for multimeric forms. As known for IgM, multimeric IgA species have an enhanced avidity, increased antigen binding and effector functions [7,8]. For example, in vitro data demonstrate a 15-fold stronger capacity of IgA dimers in neutralizing SARS-CoV-2 compared to monomeric IgA [9].

Despite its high abundancy, knowledge concerning the functional role of serum IgA is not adequate. The reasons are: (1) limitations in animal models (e.g., for rodents: missing FcαRI, polymeric IgA molecule instead of monomeric IgA in human); (2) recombinant production of IgA is difficult by the high and heterogeneous glycosylation; and (3) the low stability as well as the short serum half-life compared to IgG [5,10,11,12,13].

Intravenous immunoglobulin (IVIG) preparations contain ≥ 95% IgG with a plasma-like IgG subclass distribution and only traces of IgA and IgM [14,15]. IVIGs are used as replacement therapy for immunoglobulin deficiency in low doses (to support host immune response) or in high doses (as immune modulator) in a variety of autoimmune and inflammatory disorders [14,15,16,17].

The addition of IgM and IgA components to IVIG preparations extends the repertoire of immunoglobulins and opens promising opportunities, the first being the possibility to treat patients with IgM or IgA deficiencies and the second being the beneficial properties and functions of IgM and IgA in comparison to IgG [17,18,19]. Positive effects of IgM/IgA-enriched preparations are shown by the commercial product Pentaglobin^®^ (12% IgM, 12% IgA and 76% IgG) [18,20] and the development product trimodulin (23% IgM, 21% IgA and 56% IgG) [18]. The effectivity of trimodulin was shown in subgroups (high inflammation and/or low IgM levels) of ventilated severe community-acquired pneumonia (sCAP) patients in a clinical phase II study (CIGMA-study, NCT01420744) [21]. Trimodulin is additionally in clinical testing for treatment of hospitalized COVID-19 patients (ESsCOVID-study, NCT04576728) [22]. So far, recruitment has been completed and data are under evaluation.

Superior effects of IgM/IgA-enriched immunoglobulins compared to standard IVIG therapy were shown by several in vitro and in vivo studies in the treatment of inflammatory diseases [23,24,25,26,27,28]. Immunomodulatory (observed in high-dose therapy) as well as anti-pathogenic properties (observed in low-dose therapy) of immunoglobulin preparations can be beneficial, depending on the inflammatory status of the patient. In line with the various functions of immunoglobulins in vivo, immunoglobulin preparations induce complex and multifaceted modes of action [20,29,30,31,32,33,34,35].

Previous investigations address desirable effects on the well-known effector functions of IgG and IgM [23,25,26,27,28,36]. In contrast, some other work—focused on plasma-derived IgA—demonstrate promising anti-pathogenic and immunomodulatory effects of IgA by the interaction with FcαRI [37,38,39,40,41,42]. Nevertheless, great diversity and heterogeneity of immunoglobulin species in plasma preparations, as well as multiple modes of action, impede research.

Considering the knowns and unknowns of IgA- and IgM/IgA-enriched immunoglobulin preparations, further studies with a focus on IgA function are urgently needed. In our previous work, we showed the immunomodulatory properties of trimodulin in a neutrophil COVID-19 model. We demonstrated superior immunomodulation by trimodulin in comparison to standard IVIG and attributed these effects to the additional IgA component of trimodulin [43]. We used the neutrophil-like HL-60 cell line because neutrophils have crucial functions in immunity and are—dependent on their potent effector functions—a main inducer of tissue damage when exhausted. Furthermore, neutrophils were shown to be more strongly activated by IgA compared to IgG [44,45,46,47].

In this work, we address the functional role of IgA in trimodulin modes of action more in detail. We investigated the immunomodulatory and anti-pathogenic effects of trimodulin and IgA in context of neutrophil homeostasis and bacterial inflammation. Furthermore, we address the impact of immunoglobulin dosage on cellular effector outcome. To highlight the IgA-mediated functions on neutrophils, we compare effects mediated by IVIG, exclude FcR-dependent effects of the IgM component and focus on the interaction between trimodulin IgA and FcαRI.

## 2. Materials and Methods

### 2.1. Cell Culture

HL-60 cells (ATCC #CCL-240) were cultured in a medium containing Iscove Modified Dulbecco Media (IMDM) (Life Technologies, Carlsbad, CA, USA), 20% heat-inactivated fetal bovine serum (FBS) (Life Technologies, Carlsbad, CA, USA) and 1% Penicillin/Streptomycin (Sigma-Aldrich, St. Louis, MO, USA). Neutrophil-like phenotype was induced by centrifugation of HL-60 cells (350× *g*, 5 min) and following resuspension in described medium with 1.3% (*v*/*v*) dimethylsulfoxide (DMSO) (Sigma-Aldrich, St. Louis, MO, USA). Cells were adjusted to 6 × 10^5^ cells/mL and cultured for 4 days at 37 °C [48]. Flow cytometry analysis confirmed cell phenotype, as described in our previous work [43].

### 2.2. Immunoglobulin Preparations

In this work, the normal human IVIG preparation containing ≥ 95% IgG (IgG Next Generation, BT595, Biotest AG, Dreieich, Germany) and IgM/IgA-enriched immunoglobulin preparation with ~23% IgM, ~21% IgA and ~56% IgG (trimodulin, BT588, Biotest AG, Dreieich, Germany) were used for testing. Formulation buffer (300 mM glycine, pH 4.3) was used as a negative control; pure IgM and IgA from human serum (Sigma-Aldrich, St. Louis, MO, USA) were purchased and used for comparison.

### 2.3. Cell Treatments

We measured cytokine release and cell phenotype (FcR expression) to demonstrate direct effects of our test preparations (trimodulin and IVIG) on neutrophil-like cells. Cells were incubated for 4 h with IVIG or trimodulin at 37 °C, and IL-8 release into cell culture supernatant was analyzed (compare to Section 2.7). 

Trimodulin or IVIG was added to 300 pg/mL recombinant IL-8 in PBS to investigate direct cytokine neutralization. The IL-8 immunoglobulin mixture was incubated for 1 h at 37 °C and the remaining soluble IL-8 was measured by ELISA.

Different doses of lipopolysaccharide (LPS) were added to cells for 24 h. Cellular inflammation was determined by the release of various inflammatory cytokines via cytokine array (compare to Section 2.7) and measurement of IL-8 release was used as marker for neutrophil activation. Furthermore, modulation of FcR expression was measured (compare to Section 2.4). Influence of immunoglobulins on LPS-induced inflammation was tested by adding 15 g/L trimodulin or IVIG to LPS stimulated cells. Immunoglobulin preparations were added for another 24 h after pre-treatment of cells with LPS.

### 2.4. Analysis of FcR Expression

Fluorophore conjugated detection antibodies specific for FcR (FcαRI, FcγRI, FcγRIIA, FcγRIIB and FcγRIII) and isotype controls according to Table S1 were used. Cell viability was assessed using Zombie Aqua (BioLegend, San Diego, CA, USA) staining. For staining of 1 × 10^6^ cells, antibodies were mixed and added to cells. AF647 NHS-Ester (Thermo Fisher Scientific, Waltham, MA, USA) was used according to the manufacturer to label FcγRIIB antibodies. HL-60 cells were stained for 30 min at 4 °C and analyzed using FACS Canto II Cytometer (BD Biosciences, Franklin Lakes, NJ, USA).

### 2.5. S. aureus Phagocytosis Assay

Immune complexes (ICs) were generated using heat-inactivated *S. aureus* (BioParticles™, Alexa Fluor™ 488) (Thermo Fisher Scientific, Waltham, MA, USA). Then, 500 µL PBS was used to reconstitute one vial of *S. aureus* particles. For 30 min, 50 µg/mL immunoglobulin preparations were incubated with 5 × 10^7^ *S. aureus* particles/mL. Afterwards, the mixture was centrifuged at 1100× *g* for 18 min. The ICs were washed with PBS and resolved in 100 µL IMDM.

The *S. aureus* IC were characterized by the detection of IgG, IgA and IgM on the particle surface. Staining was performed as described above. Divergent centrifugation was performed at 1100× *g* for 18 min. As detection antibodies, anti-human IgG-APC-H7 (BD-Biosciences, Franklin Lakes, NJ, USA), IgA-VioBlue (Miltenyi Biotec, Bergisch Gladbach, Germany) and IgM-PE-Cy5 (BD Biosciences, Franklin Lakes, NJ, USA) were selected. Gates were placed using isotype controls.

Then, 1.25 × 10^6^ cells/mL were washed and resolved in IMDM without FBS. For phagocytosis, the *S. aureus* IC was mixed with neutrophil-like HL-60 cells for 1 h at 37 °C. After the phagocytosis step, cells were harvested (350× *g*, 5 min) and cell culture supernatant was stored for subsequent cytokine analysis at −20 °C. Cells were washed with PBS and extracellular fluorescence was quenched with 0.2% trypane-blue solution (Life Technologies, Carlsbad, CA, USA). Phagocytosis of *S. aureus* particles by neutrophil-like cells was measured on a FACS-Canto II cytometer. The percentage of positive cells was multiplied with the median fluorescence intensity of these cells to calculate the phagocytic index.

FcR activity was blocked using 5 µg/mL specific blocking antibodies against human FcαRI, clone MIP8α (Bio-Rad, Hercules, CA, USA); FcγRI, clone 10.1 (BioLegend, San Diego, CA, USA); FcγRIIA, clone IV.3 (StemCell, Vancouver, BC, Canada); FcγRIIB, clone 2B6 (Creative BioLabs, Shirley, NY, USA); and FcγRIII, clone 3G8 (BioLegend, San Diego, CA, USA). FcR blocking was performed 20 min before the addition of IC to cells. Cells not treated with blocking antibodies were referenced as 100% and remaining phagocytic index was calculated.

Furthermore, dose-dependent effects of trimodulin on *S. aureus* phagocytosis were investigated. Concentrations ranging from 0.05 to 20 g/L trimodulin were incubated with 5 × 10^7^ *S. aureus* particles and neutrophil-like HL-60 cells.

### 2.6. Signaling Experiments

Phosphatase SHIP-1 (ITIM) and SHP-1 (ITAMi) are central for immunoglobulin receptor signaling pathways. Inhibitors for SHIP-1 (3AC) and SHP-1 (NSC-87877) were selected to examine the importance of these signaling pathways for trimodulin and IVIG modes of action. Inhibitors were solved in DMSO and diluted 1:10 in IMDM without FBS. Thirty minutes before immunoglobulin preparations or IC, either 10 µM 3AC or 200 µM NSC-87877 were added. IL-8 release was monitored to measure inhibitor effects.

Additionally, SHP-1 activation was analyzed by SHP-1 phosphorylation at tyrosine 536 (pY536) [49]. After treatment of cells with the *S. aureus* IC and trimodulin for 90 min, fixation, permeabilization and staining with anti-SHP-1-pY536 antibody (Abwiz Bio Inc, San Diego, CA, USA) followed. IC-treated cells were used as 100% normalization control, and percentage change by trimodulin treatment was determined.

### 2.7. Measurement of Cytokine Release

Cytokine concentration in cell culture medium was quantified with human IL-8 simple step ELISA Kit (Abcam, Cambridge, UK) according to the manufacturer. Semi-quantitative comparison of different cytokines was performed with a human cytokine array kit (R&D Systems, Minneapolis, MN, USA). Divergent from the manufacturer’s, instructions membranes were stained for 30 min with IRDye-800CW Streptavidin (LI-COR, Lincoln, NE, USA) 1:2000 diluted. The Odyssey Infrared Imaging System was used for scanning membranes and the grid-array function was used to determine spot intensities.

### 2.8. Statistical Analysis

GraphPad PRISM (Version 6.1, GrapPad Software Inc, San Diego, CA, USA) was used to calculate statistics. Values are shown as mean ± standard deviation with the indicated repetitions. Two-way analysis of variance (ANOVA) with Tukey’s multiple comparisons test and one-way ANOVA with Dunnett’s multiple comparison test were performed as depicted.

## 3. Results

Antibodies mediate crucial functions to maintain the functional integrity of the human immune system. In our work, we tested the functional role of IgA and trimodulin in the context of several antibody functions: (1) facilitation of immune homeostasis, (2) immunomodulation, (3) anti-pathogenic effects and (4) immunoglobulin concentration as a critical parameter for the balance between these effector functions. Under these experimental conditions, we compared trimodulin-mediated effects with classical IVIG and focused on the interaction of IgA with FcαRI. With this approach, we want to unravel the functional role of IgA in trimodulin.

We characterized and compared the neutrophil-like HL-60 cell line to primary neutrophils in our previous work and showed that the HL-60 cell line can be used as a model for human neutrophils [43]. Important for the purpose of this work is the absence of IgM–FcµR on neutrophils and therefore the exclusion of IgM FcR-dependent effects [50].

### 3.1. Trimodulin Faciliates Immune Homeostasis

**Figure 1:**
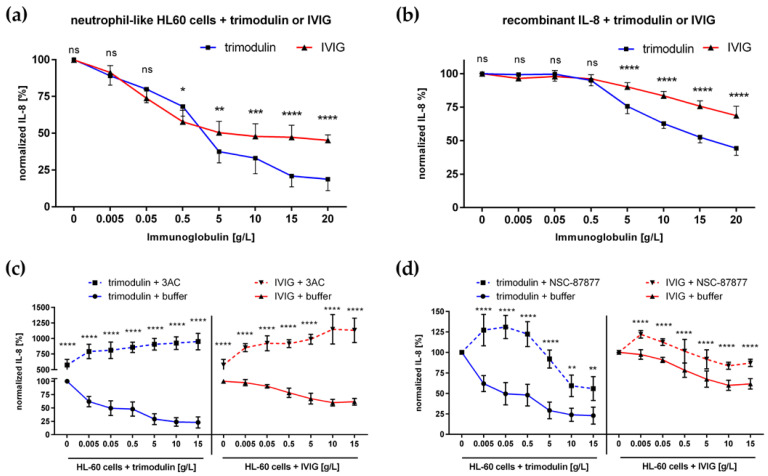
Trimodulin facilitates immune homeostasis by three modes of action. (**a**) Incubation of neutrophil-like HL-60 cells with trimodulin or IVIG reduce IL-8 secretion. Indicated concentrations of trimodulin (blue line) or IVIG (red line) were added to cells for 4 h; after centrifugation, supernatant was analyzed for IL-8 concentration via ELISA. (**b**) Trimodulin or IVIG are able to bind free IL-8 in PBS. Recombinant IL-8 was dissolved at 300 ng/mL in PBS and depicted concentrations of trimodulin (blue line) or IVIG (red line) were added; the mixture was incubated for 1 h and the remaining IL-8 was measured. (**c**) Activation of IL-8 release in correlation with blocking of ITIM signaling. 10 µM SHIP-1 inhibitor 3AC were pre-incubated with cells (30 min) and different concentrations of trimodulin or IVIG were added for 4 h. Cells were treated with depicted concentrations of trimodulin (blue lines) or IVIG (red lines). IL-8 release of cells without inhibitor 3AC (solid lines) and with inhibitor 3AC (dotted lines) was compared. (**d**) Inhibitory ITAMi signaling is activated by incubating HL-60 cells with trimodulin or IVIG. Same as in (**c**), except cells were pre-incubated with 200 µM NSC-87877 (SHP-1 Inhibitor) or not. The data are shown as mean values of six independent experiments. Statistics: Two-way ANOVA, Sidak multiple comparison test between trimodulin and IVIG group (**a**,**b**) or within trimodulin/IVIG group +/− inhibitor treatment (**c**,**d**), 95% confidence interval. * *p* ≤ 0.05, ** *p* ≤ 0.01, *** *p* ≤ 0.001, **** *p* ≤ 0.0001, ns = not significant.

In the first setting, we analyzed the general properties of immunoglobulins to facilitate immune homeostasis on neutrophil-like HL-60 cells without pathogenic stimulus. For these investigations, we focus on IL-8 release, a marker of neutrophil migration and inflammatory activation. Trimodulin or IVIG were added in several concentrations to resting HL-60 cells and IL-8 release was measured (Figure 1a). A dose-dependent reduction of IL-8 by trimodulin and IVIG was observed, with a significant stronger reduction by trimodulin in high doses > 5 g/L.

Our aim was to investigate why trimodulin could mediate a significantly stronger reduction of cytokine release. First, we prove the ability to directly interact and neutralize IL-8. As shown in Figure 1b, trimodulin can reduce the available IL-8 level in PBS significantly stronger than IVIG in doses from 5 to 20 g/L.

Next, we investigated the ability of trimodulin and IVIG to modulate inhibitory immunoreceptor tyrosine-based inhibition motif (ITIM) signaling. Blocking of central ITIM phosphatase SHIP-1 with chemical inhibitor 3AC leads to a strong increase (10-fold induction) in inflammatory IL-8 release compared to non-inhibited cells (Figure 1c).

Furthermore, trimodulin or IVIG could induce inhibitory immunoreceptor tyrosine-based activation motif (ITAMi) signaling. To verify the role of ITAMi activation, we inhibited the central phosphatase SHP-1 with inhibitor NSC-87877. Inhibition of SHP-1 increased IL-8 release from immunoglobulin-treated cells compared to uninhibited cells. Trimodulin-treated cells exhibited a stronger increase in IL-8 compared to IVIG (Figure 1d).

The modulation of FcR signaling pathways is mediated by the binding of immunoglobulins to their cellular FcR [51]. Therefore, we further tested the ability of trimodulin and IVIg to modulate FcR expression on neutrophil-like HL-60 cells. Both preparations upregulated FcγRIIB expression significantly compared to the buffer control (Figure S1c). Expression of FcαRI did not change due to IgA binding (Figure S1e). Similarly, the expression of FcγRI, FcγRIIA and FcγRIII on resting cells was not modulated due to IgG binding (Figure S1a,b,d).

### 3.2. Reduced Inflammation and Targeting of FcαRI in an Endotoxin Model

Endotoxins such as LPS are critical promotors of inflammation in sepsis [52]. In the treatment of such hyperinflammatory patients, high doses of IgM/IgA-enriched immunoglobulins were shown to be more efficient compared to classical IVIG [18,20,34]. Therefore, we hypothesized that this beneficial effect could be attributed—in part—to the IgA component of IgM/IgA-enriched preparations such as trimodulin.

**Figure 2:**
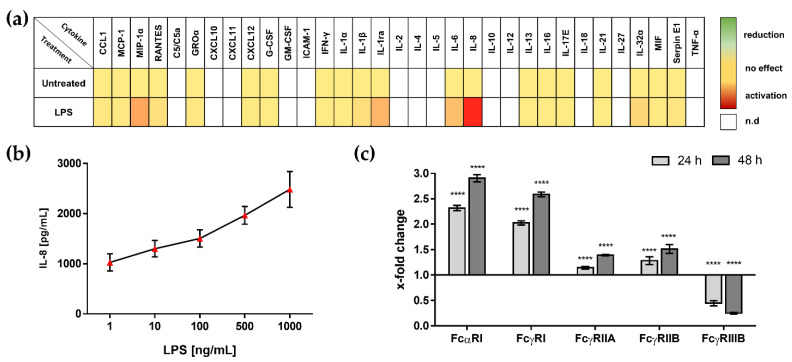
LPS-induced inflammatory phenotype of neutrophil-like HL-60 cells. (**a**) Heat map of released cytokines after treatment with LPS. HL-60 cells were incubated with 500 ng/mL LPS (18 h); supernatant of cell culture was analyzed by semi-quantitative cytokine arrays. Signal intensities of the measured cytokines of untreated cells were referenced as 1 (yellow) and the x-fold increase (red) or decrease (green) calculated. Cytokines below the detection limit are shown in white (n.d. = not detected). Data show the mean of 3 independent experiments. (**b**) IL-8 release is dose-dependently induced by LPS. Indicated concentrations of LPS were added to HL-60 cells for 24 h. IL-8 release was measured by ELISA. (**c**) LPS treatment modulates FcR expression. Neutrophil-like HL-60 cells were incubated with 500 ng/mL LPS for 24 h (light gray bars) or 48 h (dark gray bars). By immunological staining using flow cytometry, FcR expression was analyzed. The x-fold change in fluorescence value of untreated cells to treated cells was evaluated. Signal of untreated cells was set to 1 and change was calculated. Six independent experiments were performed for evaluation. Statistics: Two-way ANOVA, Sidak multiple comparison test calculated between untreated cells (baseline) and 24/48 h LPS-treated cells, 95% confidence interval. **** *p* ≤ 0.0001.

First, we characterized the LPS-induced inflammation on neutrophil-like HL-60 cells. LPS treatment lead to the release of various inflammatory cytokines (e.g., MIP1-α, IL1-β, IL-6, IL-8 or IL32a) as detected by cytokine array (Figure 2a). For further investigations, we used the strongly induced chemokine IL-8, which was dose-dependently upregulated (Figure 2b).

Modulation of FcR expression by LPS is known as FcR–TLR cross-talk [53,54]. This interaction was also observed in the neutrophil-like HL-60 cells. LPS stimulation enhanced expression of FcαRI, FcγRI, FcγRIIA and FcγRIIB and decreased expression of FcγRIII (Figure 2c).

Next, we investigated the immunomodulatory effects of trimodulin and IVIG on inflammatory neutrophil-like HL-60 cells stimulated with LPS. We added different doses of both preparations to LPS pre-stimulated cells and measured IL-8 release as well as modulation of FcR expression.

**Figure 3:**
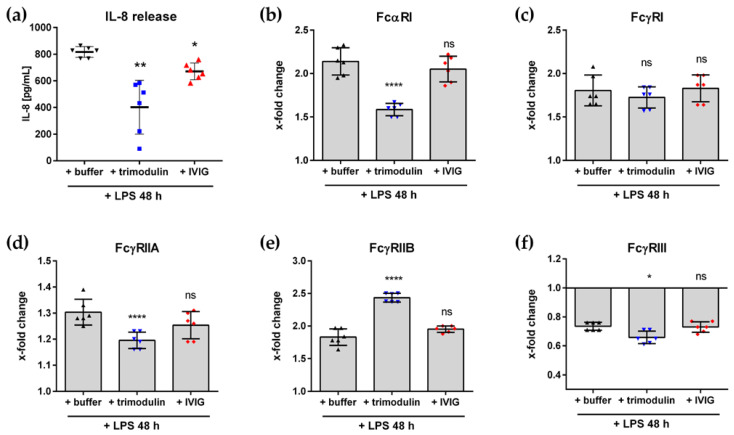
Modulation of LPS-induced cytokine release and FcR expression by trimodulin and IVIG. (**a**) Addition of trimodulin or IVIG decrease LPS-induced IL-8 secretion. Neutrophil-like HL-60 cells were treated with 500 ng/mL LPS for 48 h and in the following 24 h, with 15 g/L trimodulin (blue points), IVIG (red points) or equal volume buffer (black points). Via ELISA, the IL-8 level in the cell culture supernatant was analyzed. (**b**–**f**) Modulation of LPS-induced FcR expression. Cells were incubated with LPS (48 h; 500 ng/mL) and afterwards, with 15 g/L trimodulin, IVIG or equal volume buffer (for 24 h). FcR expression was examined by flow cytometry. Measured fluorescence value of LPS-treated cells was referenced as 1 (baseline) and x-fold change after immunoglobulin treatment was determined. The mean of 6 independent experiments is depicted. Statistics: One way ANOVA; Dunnett’s multiple comparisons test calculated between buffer control and trimodulin or IVIG, 95% confidence interval. * *p* ≤ 0.05, ** *p* ≤ 0.01, **** *p* ≤ 0.0001, ns = not significant.

Similar to resting cells, trimodulin and IVIG reduced inflammatory cytokine release dose-dependently, with a significant stronger reduction of IL-8 in high doses of trimodulin compared to IVIG (Figure S2 and Figure 3a).

In addition, the cellular phenotype (depicted as FcR expression) was especially modulated by trimodulin. Detection of FcαRI, FcγRIIA and FcγRIII was significant reduced by trimodulin compared to the buffer control (Figure 3b,d,f). FcγRI was not affected by both preparations (Figure 3c) and FcγRIIB was significantly upregulated by trimodulin (Figure 3e). The treatment with classical IVIG did not significantly affect the detection of FcR. The reduction of FcαRI expression with trimodulin shows that the IgA component of trimodulin directly interacts with its cellular FcR, thereby shaping cellular phenotype. In sum, we showed superior modulation of cytokine release and FcR expression by trimodulin, as well as the involvement of IgA in trimodulin-mediated immunomodulation.

### 3.3. Trimodulin IgA Mediates FcαRI-Dependent Phagocytosis of S. aureus

In the third part of this work, we investigate the anti-pathogenic effects of the trimodulin IgA component by analyzing opsonization and phagocytosis of *S. aureus*.

**Figure 4:**
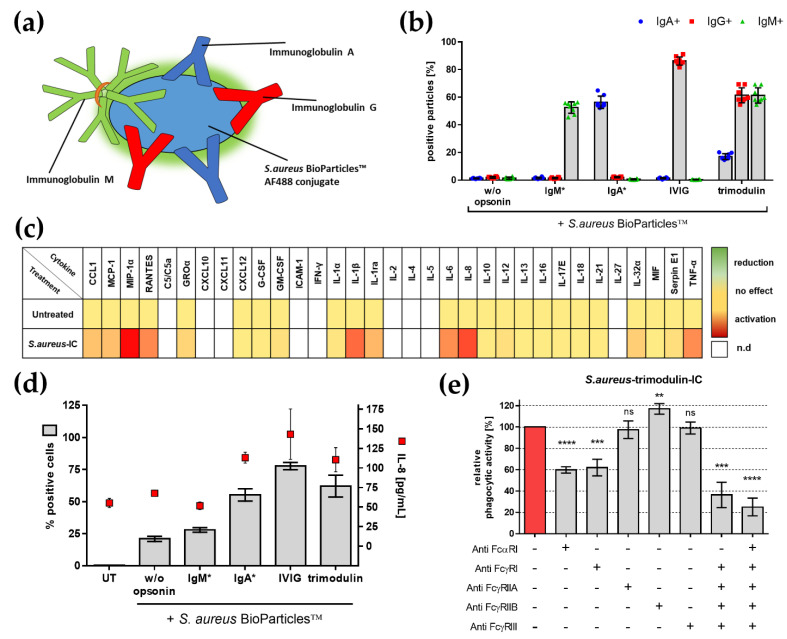
Antibody-dependent phagocytosis of *S. aureus*. (**a**) Schematic overview of the *S. aureus*–trimodulin immune complex (IC). (**b**) *S. aureus* particles were bound by IgM, IgA and IgG species. Binding of immunoglobulins to *S. aureus* bioparticles after 30 min of incubation were detected using specific detection antibodies. Percentage of IgM (green triangle), IgA (blue dots) and IgG (red square) on positive *S. aureus* particles was determined by flow cytometry. * Purified IgA and IgM from human serum. (**c**) Cytokine heat map after stimulation of HL-60 cells with *S. aureus*–IVIG IC. Neutrophil-like HL-60 cells were stimulated with *S. aureus*–IVIG IC (18 h); supernatant of cell culture was analyzed by semi-quantitative cytokine arrays. Signal intensities of cytokines measured by untreated cells were referenced as 1 (yellow) and the x-fold increase (red) or decrease (green) was calculated. Cytokines below the detection limit are shown in white (n.d. = not detected). Data show the mean of 3 independent experiments. (**d**) Phagocytosis of *S. aureus* bioparticles with different immunoglobulin preparations; 50 µg/mL immunoglobulin preparations or buffer were added for opsonization to *S. aureus* particles for 1 h. Phagocytosis was measured as percentage fluorescence positive cells (gray bars). Corresponding IL-8 release was analyzed by ELISA (red dots). * As controls, purified IgA and IgM from human serum was used. (**e**) FcR blocking experiments with *S. aureus*–trimodulin IC. Blocking antibodies (5 µg/mL) were added 20 min before *S. aureus*–trimodulin IC to cells. Phagocytic index (percentage positive cells multiplied with median fluorescence intensity) of non-blocked cells was referenced as 100% and remaining phagocytosis was calculated. Values represent mean of 6 independent experiments. Statistics: One way ANOVA; Dunnett’s multiple comparisons test between non-blocked cells (100% control) and treatment with indicated blocking antibodies, 95% confidence interval. * *p* ≤ 0.05, ** *p* ≤ 0.01, *** *p* ≤ 0.001, **** *p* ≤ 0.0001, ns = not significant.

We generated immune complexes (IC) by opsonization of heat-inactivated and fluorophore-labeled *S. aureus* particles with different immunoglobulin preparations (Figure 4a). We detected IgG, IgA and IgM antibodies bound on *S. aureus* particles via flow cytometry to unravel which class is relevant for opsonization. The IC generated with pure commercial IgM or IgA, as well as IgG containing IVIG, depicted sole binding of the corresponding immunoglobulin class. In contrast, the trimodulin IC showed binding of IgG, IgA and IgM on *S. aureus* bioparticles (Figure 4b).

Next, the *S. aureus* IC was added to neutrophil-like HL-60 cells for subsequent phagocytosis and induction of inflammation. The addition of the *S. aureus* IC led to inflammatory activation and release of pro-inflammatory cytokines. Compared to untreated cells, increased levels MCP-1, MIP-1α, RANTES, IL-1β, IL-6, IL-8, IL32a and TNFα were observed (Figure 4c).

Besides cytokine release, we measured phagocytosis as uptake of fluorescent *S. aureus* particles or IC. In the control sample with non-opsonized *S. aureus* particles, ~20% of cells phagocytose *S. aureus*; similar levels were observed for IgM-opsonized particles. In contrast, IgA, IVIG or trimodulin *S. aureus* IC-enhanced phagocytosis and corresponding IL-8 release (Figure 4d).

To verify the functional role of IgG and IgA on trimodulin IC phagocytosis, we performed FcR blocking experiments. Blocking of FcαRI, FcγRI or their combinations significantly reduced phagocytosis. Contrarily, blocking of FcγRIIB enhanced phagocytosis (Figure 4e). FcR blocking of the IgA IC showed a full FcαRI-dependent phagocytosis. IVIG IC uptake was FcγRI- and FcγRIIA-dependent, whereas non-opsonized *S. aureus* particles showed a significant reduction of phagocytosis when FcαRI was blocked (Figure S3).

Summarized, these data show that specific antibodies of IgM, IgA and IgG classes in trimodulin bind and opsonize *S. aureus* surface antigens. Functionally important for neutrophil-mediated ADCP are IgG and IgA species. These results clearly demonstrate the anti-pathogenic role of the trimodulin IgA component against *S. aureus*.

### 3.4. Dose-Dependent Function of Trimodulin

As mentioned above, immunoglobulin preparations were used in different dosages, dependent on the immunological status of the patient [14,16,55].

**Figure 5:**
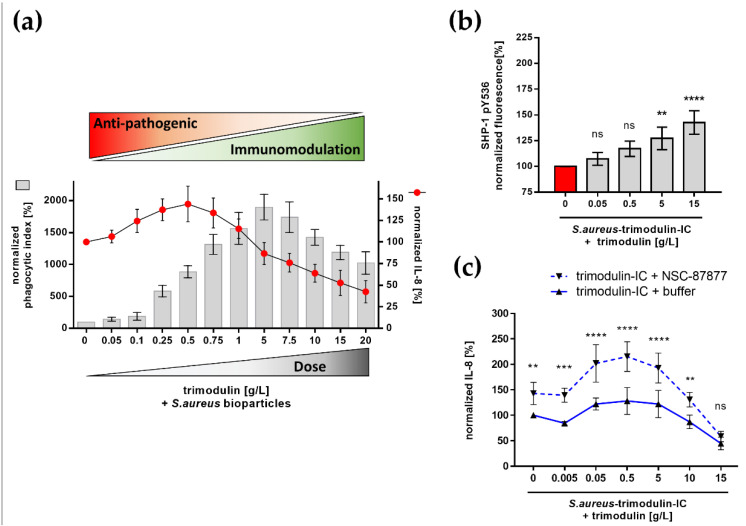
Dose-dependent effects of trimodulin on *S. aureus* phagocytosis. (**a**) Dose dependency of trimodulin on phagocytosis of *S. aureus*. Indicated concentrations of trimodulin were added with *S. aureus* bioparticles for 1 h to neutrophil-like HL-60 cells. Phagocytosis (gray bars) and corresponding IL-8 release (red dots) are shown. Low doses of trimodulin mediate anti-pathogenic effects, as shown by increased phagocytosis and cytokine release. Higher doses mediate immunomodulatory effects with reduced phagocytosis and inflammation. (**b**) ITAMi signaling induced by high concentrations of trimodulin. Tyrosine 536 (pY536) phosphorylation of phosphatase SHP-1 was measured. Therefore, *S. aureus*–trimodulin IC and depicted trimodulin concentrations were added to cells for 90 min. Intracellular staining with anti-phospho-SHP-1 pY536 antibody was performed. Fluorescence was normalized to buffer control. (**c**) SHP-1 important for immunomodulation. Cell treatment as in (**b**), with additional 30 min of pre-incubation with 200 µM NSC-87877. IL-8 release between NSC-87877 (dotted lines) or buffer treatment (solid lines) is shown. Values represent mean of 6 independent experiments. Statistics: Two-way ANOVA; Tukey’s multiple comparisons test between *S. aureus*–trimodulin IC control (100%) and trimodulin addition (**b**) or within trimodulin + buffer and trimodulin + NSC-87877 treatment (**c**). * *p* ≤ 0.05, ** *p* ≤ 0.01, *** *p* ≤ 0.001, **** *p* ≤ 0.0001, ns = not significant.

Therefore, we sought to investigate dose-dependent effects of trimodulin on neutrophil-like HL-60 cells. We added several concentrations of trimodulin, ranging from 0.05 to 20 g/L, to *S. aureus* particles, then measured subsequent phagocytosis and IL-8 release. In low doses (up to 5 g/L), we observed anti-pathogenic effects, seen by increasing levels of phagocytosis and IL-8. Higher doses (above 5 g/L) lead to decreasing phagocytosis and IL-8 release (Figure 5a).

Mechanistically decreasing levels of phagocytosis reveal a displacement of IC from activating FcR by an excess of immunoglobulins. Furthermore, these high levels of circulating immunoglobulins could activate inhibitory ITAMi signaling as an additional mode of action. In line with this, we demonstrated activation of ITAMi in high doses of trimodulin by a significant phosphorylation of SHP-1 (Figure 5b). Moreover, blocking of SHP-1 by chemical inhibitor NSC-87877 lead to a significantly increased inflammatory IL-8 release, which confirmed the anti-inflammatory effects of ITAMi (Figure 5c).

Trimodulin mediates dose-dependent effects on neutrophil-like HL-60 cells. As reported in the literature, low doses induce anti-pathogenic effects, whereas higher doses induce immunomodulation. This process was shown to be dependent on displacement of IC and activation of inhibitory ITAMi signaling.

## 4. Discussion

Despite its high abundancy, human serum IgA is not well investigated in comparison to IgG [5,10,12]. Similarly, the role of IgA in IgM/IgA-enriched immunoglobulin preparations is not yet fully unraveled. The heterogeneity and complexity of these preparations makes a separation of the three components challenging, which hampers research. Nevertheless, several studies focusing on IgA showed the powerful properties of this immunoglobulin class. IgA mediates stronger activation of ADCC on neutrophils and ADCP on macrophages than IgG. In particular, the IgA-mediated activation of neutrophils could be a powerful tool in cancer therapy [11,44,56,57,58,59]. On the other hand, overwhelming inflammation by neutrophils is a harmful factor for tissue damage in severe infectious diseases [60,61,62]. In this case, the anti-inflammatory properties of IgA could mediate powerful immunomodulation [39,63,64,65,66,67,68,69,70].

To shed light on these manifold functions of IgA in trimodulin, we used several experimental settings: (1) the anti-inflammatory effects on resting neutrophils and maintenance of immune homeostasis; (2) immunomodulation in an endotoxin model of hyperinflammation; (3) anti-pathogenic effects against *S. aureus*; and (4) the importance of immunoglobulin dosage for the balance between anti-pathogenic and immunomodulatory effector outcomes.

### 4.1. The Maintanance of Immune Homeostasis

Immune homeostasis is regulated by the interaction of monomeric immunoglobulins with resting or activated immune cells. The low affinity binding of monomeric immunoglobulin IgG or IgA on FcR mediates inhibitory signaling and this induces anti-inflammatory effects during homeostasis [54,71,72]. The addition of trimodulin and IVIG to resting neutrophil-like HL-60 cells drives the reduction of inflammatory cytokines and facilitates an anti-inflammatory phenotype (Figure 1). Our data reveal that three synergistic modes of action facilitate an anti-inflammatory phenotype and immune homeostasis: (1) the direct neutralization of cytokines; (2) upregulation of inhibitory FcγRIIB, as well as induction of ITIM signaling; and (3) activation of inhibitory ITAMi signaling.

Trimodulin was able to induce an anti-inflammatory phenotype better than IVIG. The reason is the stronger direct binding and neutralization of IL-8, as shown for activated complement components [26]. In particular, the multimeric IgA and IgM species in trimodulin could mediate multivalent binding and thereby reduce levels of inflammatory cytokines. Furthermore, trimodulin induced inhibitory ITAMi signaling more strongly compared to classical IVIG. The reason could be the simultaneous targeting of IgA and IgG FcR. Additionally, stronger ITAMi signaling could be induced due to 2:1 stoichiometry by IgA–FcαRI binding, whereas IgG binds FcγR in 1:1 stoichiometry [44,73].

Despite the fact that trimodulin has a much lower IgG portion (~56% IgG) than IVIG (≥95% IgG), both preparations similarly induced expression of inhibitory FcγRIIB, as well as inhibitory ITIM signaling. IVIG preparations are known to modulate the balance between activating FcγR and inhibitory FcγRIIB by upregulation of FcγRIIB [18,34,74]. The crucial role of ITIM signaling in maintaining immune cell homeostasis was shown by inhibiting SHIP-1 phosphatase [72]. Inhibition of SHIP-1 strongly enhanced IL-8 release of neutrophil-like HL-60 cells treated with immunoglobulin preparations. In contrast to inhibitory FcγRIIB, neither activating FcαRI nor FcγR were modulated by both immunoglobulin preparations. Low affinity binding of monomeric IgG and IgA under resting conditions could not be sufficient to induce FcR internalization [10,75,76,77].

Summarized, trimodulin has superior functions in maintaining immune homeostasis of neutrophils; the additional IgA component is a reason for the beneficial effects in comparison to standard IVIG.

### 4.2. The Immunomodulation by IgA–FcαRI Axis in an Endotoxin Model

In severe infectious diseases, systemic inflammation and mortality are often facilitated by bacterial endotoxins. Destruction of bacterial cells by antibiotic treatment increases endotoxin load and forces inflammation [78,79]. Therefore, we stimulated our neutrophil-like HL-60 cell model with endotoxin (using LPS derived from *E.coli*), characterized the inflammatory effects (Figure 2) and evaluated the immunomodulatory properties of our immunoglobulin preparations in this kind of inflammation (Figure 3).

The release of pro-inflammatory cytokines by neutrophil-like HL-60 cells stimulated with LPS is dose-dependent. The observed cytokines correlate to inflammatory markers as predictors for sepsis severity and mortality (e.g., IL-6, IL-8, MIP-1α or IL1ra) [52,80]. Induction of inflammation is mediated by the binding of LPS to toll-like-receptor 4 (TLR 4) and corresponding TLR signaling (data not shown) [20,53,81].

The addition of LPS to neutrophil-like HL-60 cells induced upregulation of FcR expression, as reported in literature by FcR–TLR cross-talk [70,77,82,83]. The recognition of endotoxins by innate immune receptors such as TLR increases the amount of FcR per cell, priming the cell for adaptive immunity (e.g., the neutralization of antibody opsonized pathogens) [54]. As seen for co-stimulation of IgA and IgG ICs with LPS, FcR–TLR cross-talk induces potent inflammation [37,84]. This demonstrates the complexity and interplay between differing receptors and signaling cascades.

In contrast to the other activating FcR, FcγRIII detection was reduced after LPS treatment. This observation could be attributed to the proteolytic cleavage of FcγRIIIB by metalloprotease A disintegrin and metalloprotease 17 (ADAM17). The occurring soluble FcγRIIIB promotes inflammation by interaction with other immune cells and the induction of pro-inflammatory cytokines release [85,86]. In summary, LPS treatment induces an inflammatory phenotype of neutrophil-like HL-60 cells. Dose-dependent changes in FcR expression and increased release of inflammatory cytokines were observed.

The treatment with high doses of immunoglobulin preparations is a promising adjunctive therapy to modulate an overwhelming immune system. Compared to classical IVIG, IgM/IgA-enriched immunoglobulins were shown to be more efficient in treating inflammatory sepsis patients [18,20,34]. We hypothesized that the beneficial immunomodulation could be partially attributed to the interaction of IgA with FcαRI.

To prove our hypothesis, we added trimodulin or IVIG to our inflammation model (Figure 3). The strong reduction of inflammatory IL-8 by trimodulin could explain beneficial effects for patients with severe infectious diseases, as IL-8 is of major importance for migration, inflammatory activation (e.g., induction of NETs) and tissue damage induced by neutrophils [61,62,80,87].

Besides cytokine release, LPS-induced inflammation leads to enhanced FcR expression (Figure 2). The addition of trimodulin to LPS pre-stimulated HL-60 cells counteracted these LPS-induced phenotypes. The expressions of FcαRI, FcγRIIA and FcγRIII are reduced by trimodulin. In contrast, the inhibitory FcγRIIB was upregulated. The addition of IVIG did not induce such modulations. The positive effect of trimodulin can be attributed to the IgA antibodies, which bind to FcαRI and lead to a reduced availability of FcαRI on the cell surface. Receptor aggregation and internalization is induced by the binding of monomeric and multimeric forms of IgA to FcαRI [70,88,89]. These data illustrate for the first time the meaning of FcαRI as target and the significance of the IgA–FcαRI axis for trimodulin modes of action, which does not exist in IVIGs.

IVIGs are known to shift the expression of activating FcγRIIA and inhibitory FcγRIIB [16,18,34,74,77]. In our experiments, trimodulin reduced activating FcγRIIA and elevated inhibitory FcγRIIB more strongly compared to IVIG. A differing IgG subclass distribution of the products could explain these differences: IgG1–IgG4 have divergent binding affinities to FcγR and conduct varying effector functions [90,91]. IgG subclass distribution of classical IVIGs is similar to serum source [74], whereas trimodulin has a higher portion of IgG4 (in house data). The relatively high affinity of IgG4 to inhibitory FcγRIIB could be a reason for enhanced modulation by trimodulin [91]. Furthermore, the multimeric species of trimodulin could have a better ability to interact with low affinity receptors, thereby inducing receptor internalization.

Similar to the anti-inflammatory effects on resting neutrophil-like HL-60 cells, trimodulin was able to reduce the LPS-induced cytokine release and FcR expression more efficient than classical IVIG. In line with our hypothesis, we demonstrated an important role of IgA and a more potent immunomodulation compared to IVIG. Nevertheless, the superior effects of IgM/IgA-enriched immunoglobulins compared to IVIG seem to be more complex, involving synergistic effects between the IgA–FcαRI-axis, differences in IgG subclass distribution and multimeric immunoglobulin species.

### 4.3. The Anti-Pathogenic Effects of IgA in Trimodulin

The anti-pathogenic effects of immunoglobulins are multifaceted and dependent on antibody isotype and immune effector cell. ADCP is an important mode of action in antibody-dependent pathogen clearance. The binding of IgG and IgA to pathogens mediates opsonization and subsequent phagocytosis by neutrophils [60]. For immunoglobulin preparations, the direct mediation of ADCP and induction of potent inflammatory activation was demonstrated [34,92].

In this work, the anti-pathogenic effects of IgA in trimodulin were explored by investigating the phagocytosis of *S. aureus*. This widespread, Gram-positive bacterium is the foremost pathogen in many infectious diseases, including pneumonia and sepsis [93,94,95,96]. In COVID-19 disease, co-infection with *S. aureus* is coherent with worse clinical outcomes [97,98,99,100,101]. Defeating *S. aureus* is a challenging topic in public health due to several mechanisms of the bacterium to evade the innate immune response and antibiotics [93,94].

The phagocytic properties of the trimodulin IgA component were investigated by comprehensive characterization and comparison with pure IgG, IgA and IgM preparations (Figure 4 and Figure S3). We checked which immunoglobulin classes opsonize *S. aureus* and measured phagocytosis and inflammatory cytokine release of different ICs. Furthermore, we demonstrated FcR dependency by FcR blocking experiments. These data give comprehensive insight into the process of ADCP and reveal the functional relevance of the trimodulin IgA component.

The data show direct phagocytosis of *S. aureus* in absence of opsonins, which can be explained by phagocytosis via innate immune receptors (e.g., TLR) independent of opsonins, a process called opsonin-independent phagocytosis [95,102].

Nevertheless, phagocytosis was strongly enhanced by specific opsonization of *S. aureus* with IgG and IgA. The broad spectrum of antibody specificities in the donor plasma results in immunoglobulin preparations with multiple antibodies against various pathogens (such as *S. aureus*) [15,17,20]. Secretion of several pro-inflammatory cytokines such as IL-1β, IL-6, IL-8, IL-32a, MCP-1, MIP-1α, RANTES and TNFα demonstrates phagocytosis-induced inflammation. Inflammatory activation of neutrophils by IC is known to be induced by FcR–ITAM signaling with the central SYK kinase [49,103].

Although specific IgM antibodies of the pure IgM preparation bind to the surface of *S. aureus* particles, phagocytosis was comparable to non-opsonized *S. aureus*. Absent phagocytic capacity can be justified by the missing IgM–FcµR on neutrophil-like HL-60 cells and complement factors in this assay setup [50,104].

In trimodulin, specific IgG, IgA and IgM antibodies bind to the surface of *S. aureus* and lead to opsonization; this can be attributed to the neutralizing antibody titers of each immunoglobulin class in IgM/IgA-enriched immunoglobulins ([96] and in house data). In our system, the trimodulin IC is phagocytosed via FcαRI, FcγRI or combinations of both. Despite the lower concentration of IgA on the trimodulin IC (compared to IgG and IgM), the results of blocking experiments highlight the importance of IgA for phagocytosis. An explanation for the observed stronger ADCP might be the interaction of IgA with two FcαRI molecules, thereby mediating stronger ADCP compared to IgG [11,44,45]. These data show for the first time the anti-pathogenic activity of the trimodulin IgA component.

Phagocytic activity of trimodulin opsonized *S. aureus* is lower compared to IVIG. The reason could be the negative influence of trimodulin IgM species on phagocytosis, because IgM covers binding sites with high avidity [3] and therefore hampers IgG- or IgA-mediated phagocytosis. A further reason could be the higher portion of IgG4 in trimodulin, which has a lower ability to induce effector functions such as ADCP compared to IgG1 or IgG3 [91,105,106].

FcγRIIB is known for its inhibitory effects on cellular effector functions by ITIM signaling [54,72]. Compared to activating FcαRI or FcγRs, blocking of inhibitory FcγRIIB enhances phagocytosis of IgG-containing immunoglobulin preparations, which emphasizes the important role of this receptor for immune cell homeostasis.

Summarized, the results shed light on trimodulin-mediated phagocytosis and they unravel in detail which immunoglobulin species as well as FcR are responsible for immunoglobulin–FcR interaction [107,108,109]. The experiments extend the scientific knowledge and clearly show the role of IgA in phagocytosis. An active component, IgA is an important player in the modes of action of trimodulin.

### 4.4. The Dose Dependency

In general, the antibody to antigen ratio shapes the immune response. With an optimal ratio of antibodies to antigens, optimal cross-linking of IC is achieved. A further increase in antibody concentration blocks FcR and disrupts IC formation, which consequently down-modulates the immune response [92]. This is an important step to prevent excessive inflammation and maintain immune homeostasis [75,86].

In line with this basic concept of immunology, the dosage of IVIG can be used to control effector outcomes in patients: anti-pathogenic activity is observed at low doses, whereas immunomodulation appears at high doses [14,16].

We explored the potential dual function of trimodulin by investigating the inflammatory cell activation due to phagocytosis of *S. aureus* (Figure 5). The observed data show ascending phagocytosis and correlating IL-8 levels. The IL-8 level increased with low concentrations of trimodulin up to a peak value and decreased with declining phagocytic activity induced by high concentrations of trimodulin. These immunomodulatory effects observed in our in vitro system are in accordance with clinical observations. Immunomodulation by high doses (1–3 g/kg body weight) of immunoglobulin is used in the therapy of patients with inflammatory disorder or autoimmune diseases [14,15,16,55,74,110].

In a previous work of our team, Schmidt et al. [111] compared assay concentrations with data from trimodulin clinical phase I [112] and phase II studies [21]. It became clear that the assay concentrations correspond to immunoglobulin plasma levels of healthy human subjects (~20 g/L immunoglobulin), as well as sCAP patients (~18 g/L immunoglobulin) after trimodulin therapy. In our experimental setting, we observed immunomodulatory effects in similar doses. Therefore, the in vitro data substantiate clinical results.

The in vitro system of this work has the opportunity to investigate underlying modes of action for immunomodulation. Therefore, we tested activation of inhibitory ITAMi after high-dose trimodulin treatment by monitoring the site-specific phosphorylation of ITAMi phosphatase SHP-1 and inhibiting SHP-1 activation (Figure 5b,c). The phosphorylation of SHP-1 was dose-dependent, and the specific tyrosine 536 phosphorylation of SHP-1 shows the activation of ITAMi pathway by trimodulin [49]. To confirm the importance of ITAMi, a chemical inhibitor for SHP-1 NSC-87877 was used [113] and IL-8 levels were analyzed. The increased IL-8 levels in comparison to non-inhibited cells verify the anti-inflammatory ITAMi effects. These findings demonstrate that monomeric IgG and IgA species of trimodulin lead to cell inhibition by activation of ITAMi, as previously described [63,72,107].

Reduced phagocytosis in high doses of trimodulin indicates blocking of activating FcR and displacement of IC, as additional modes of action. Less pro-inflammatory ITAM signaling is mediated by reduced phagocytosis [16,74]. IC displacement by multimeric and highly avid IgM and IgA species in trimodulin was anticipated [34,38].

To sum up, a dose-dependent function of trimodulin was shown in this work. As demonstrated by our data, the IgA component of trimodulin is involved in anti-pathogenic effects (enhanced phagocytosis) as well as in immunomodulatory effects (activation of ITAMi via FcαRI and displacement of IC).

Notably, the analyzed effector outcome (e.g., phagocytosis or cytokine release) is a shifting cell-based effect of immunomodulatory and inflammatory stimuli [54]. This balance can be modulated by treatment with immunoglobulin preparations. The results of this study show that with high dosages of trimodulin, the cell system can be shifted to immunomodulation, enabling immune homeostatic conditions.

As the in vitro data indicate, it is important to monitor the immune status of the patient for optimal immunoglobulin therapy: in cases of severe infectious diseases, patients suffer from hyperinflammation (with the need for immunomodulation and high-dose therapy), or can shift to immune suppression (with the need for immune activation and low-dose therapy) [16,20,114].

## 5. Conclusions

Our work shows the functional aspects of IgA in trimodulin modes of action. Compared to standard IVIG, the interactions between IgA and FcR/FcαRI might be a valuable benefit of trimodulin in the treatment of inflammatory diseases. Besides the important role of IgA, we demonstrate that the IgG and IgM components of trimodulin are important for trimodulin function. IgG and IgM mediate equal or differing functions as IgA.

The changes in cellular effector outcomes were mediated by a combination of several modes of action. Comparing reviewed modes of action for classical IVIG, the interplay between the three classes and the sum of effects mediated by each immunoglobulin class promote the beneficial effects of trimodulin. The investigated modes of action, the importance of the involved immunoglobulin classes and the comparison between trimodulin and IVIG are summarized in Table 1.

The multiplicity of modes of action mediated by trimodulin opens new therapeutic possibilities. In severe infectious diseases, the whole immune system is exhausted [79,115]. In these cases, the targeting of several pathways and receptors with IgG, IgA and IgM of trimodulin could be beneficial, compared to targeting only a single pathway.

As pronounced in our manuscript, a key point for successful immunoglobulin therapy is the right dosage at the right time point. High doses are required in hyperinflammatory patients, whereas low doses help immune suppressed patients. Therefore, monitoring the immunological status of the patient must be a central aspect before applying immunoglobulin preparations.

Particularly, in the focus of respiratory diseases such as sCAP or COVID-19, the modulation of neutrophils by IgA could be a promising strategy [6,12,116]. Although several authors state that IgA seems to be an important therapeutic molecule [12,44,117,118], comprehensive studies analyzing therapeutic IgA molecules are lacking. In addition, in most infectious diseases, neither levels of IgA nor the functional roles were consistently analyzed; therefore, further studies to understand the role of IgA are essential.

As addressed throughout our manuscript, the human immune system is a complex network of many interacting immune cell types and proteins [119,120]. The neutrophil in vitro models in this work cannot depict this complexity. Further work with more immune cell types, inflammatory stimuli and cellular effector outcomes is necessary to confirm and better understand the functional role of IgA in IgM/IgA-enriched immunoglobulin preparations.

## Figures and Tables

**Table 1:** Immunomodulatory modes of action mediated by trimodulin and IVIG. Experimental parameters to prove each mode of action are listed. The impacts mediated by IgG, IgA and IgM are depicted as followed: +++ strong impact, ++ medium impact, + low impact, − no impact, n/a not applicable for neutrophils.

Mode of Action	Experimental Parameter	Trimodulin			IVIG
		IgG	IgA	IgM	IgG
**(1) Direct neutralization**	Binding to IL-8 (competitive ELISA)	+	++	+++	+
**(2) ITAMi signaling**	Inhibition of SHP-1 Phosphorylation of SHP-1 at pY536	+	+++	n/a	+
**(3) ITIM signaling**	Inhibition of SHIP-1; Expression /Blocking of FcγRIIB	+++	−	−	++
**(4) Phenotype modulation**	Expression of FcαRI, FcγRI, FcγRIIA, FcγRIII	++	++	n/a	−
**(5) Phagocytosis**	Uptake of *S. aureus*, IL-8 induction	+++	++	n/a	+++
**(6) Displacement of IC**	Reduced phagocytosis	++	++	+	++

## Data Availability

Data of this study can be found in the Ph.D. thesis with the title “The immunomodulatory properties of the IgM/IgA-enriched immunoglobulin preparation trimodulin”, which was submitted to the Technical University of Darmstadt, Germany by F.B. (https://doi.org/10.26083/tuprints-00019101, accessed on 4 October 2021).

## Supplementary Materials

The following are available online at https://www.mdpi.com/article/10.3390/biomedicines9121828/s1, Figure S1: Modulation of FcR expression on resting neutrophil-like HL-60 cells, Figure S2: LPS-induced IL-8 release is reduced by the addition of trimodulin and IVIG. Figure S3: FcR blocking experiments (**a**) with *S. aureus* particles, (**b**) *S. aureus*–IgA IC (**c**) and *S. aureus*–IVIg IC. Table S1: Antibodies used in flow cytometry for immunological staining of Fc receptors on neutrophil-like HL-60 cells.
