# Supplementary material for: The Functional Role of IgA in the IgM/IgA-Enriched Immunoglobulin Preparation Trimodulin
Source: Biomedicines. 2021 Dec 3;9(12):1828. doi: 10.3390/biomedicines9121828 (PMC8698729; doi:10.3390/biomedicines9121828)
Supplement: Supplementary file 1 [file biomedicines-09-01828-s001.zip › biomedicines-1462541-SI.pdf]

<u>**Supplementary Material**</u>

Table S1. Antibodies used in flow cytometry for immunological staining of Fc-receptors on neutrophil-like HL-60 cells.

**Antigen**	**Mouse anti-human clone**	**Fluorophore**	**Manufacturer #Art. no.**	**Used volume**
FcγRI	10.1	PE-Cy7	BD Biosciences #561191	5 μL
FcγRIIA	IV.3	FITC	StemCell #60012FI	20 μL
FcγRIIB	2B6	AF647	Creative BioLabs #TAB-036WM	2.5 μL
FcγRIII	3G8	APC-Cy7	BD Biosciences #557758	5 μL
FcαRI	A59	BV421	BD Biosciences #744374	3 μL

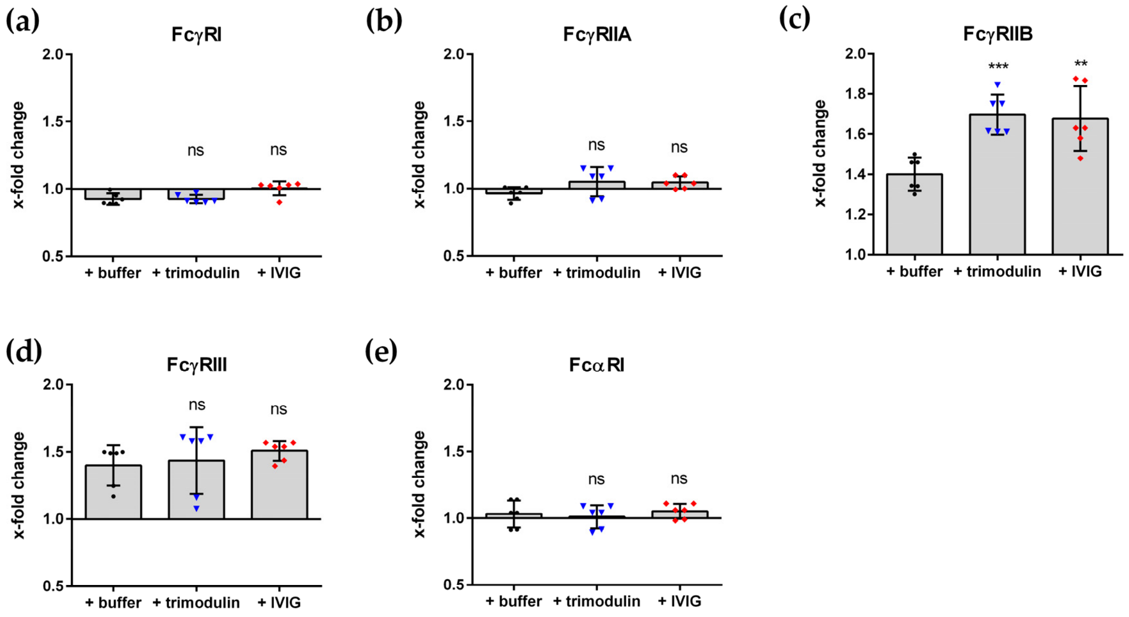

Figure S1. Modulation of FcR expression on resting neutrophil-like HL-60 cells. (**a**) Modulation of FcγRI. Neutrophil-like HL-60 cells were incubated for 24 h with 15 g/L trimodulin, IVIg or buffer. FcγRI expression was analyzed via flow cytometry. The fluorescence value of untreated cells was set as 1 and x-fold change during treatment was calculated. (**b**) Modulation of FcγRIIA. (**c**) Modulation of FcγRIIB (**d**) Modulation of FcγRIII. (**e**) Modulation of FcαRI. Values represent the mean of 6 independent experiments. Statistics: One way ANOVA; Dunnett's multiple comparisons test between buffer and trimodulin/IVIG. 95% confidence interval, * $p \leq 0.05$, ** $p \leq 0.01$, *** $p \leq 0.001$, **** $p \leq 0.0001$, ns = not significant.

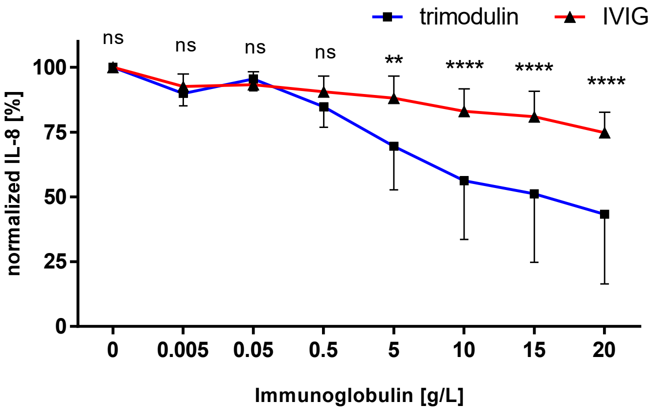

Figure S2. LPS induced IL-8 release is reduced by the addition of trimodulin and IVIG. HL-60 cells were incubated for 48 h at 37 °C with 500 ng/mL LPS and subsequently, 24 h with indicated concentrations of trimodulin (square, blue line) or IVIg (triangle, red line). Cell culture supernatant was analyzed for IL-8 release by ELISA-Kit, cells incubated with buffer were referenced as 100 % control and remaining IL-8 [%] was calculated. Values represent the mean of 6 independent experiments. Statistics: Two-way ANOVA; Tukey's multiple comparisons test between trimodulin and IVIG group. 95% confidence interval, * $p \leq 0.05$, ** $p \leq 0.01$, *** $p \leq 0.001$, **** $p \leq 0.0001$, ns = not significant.

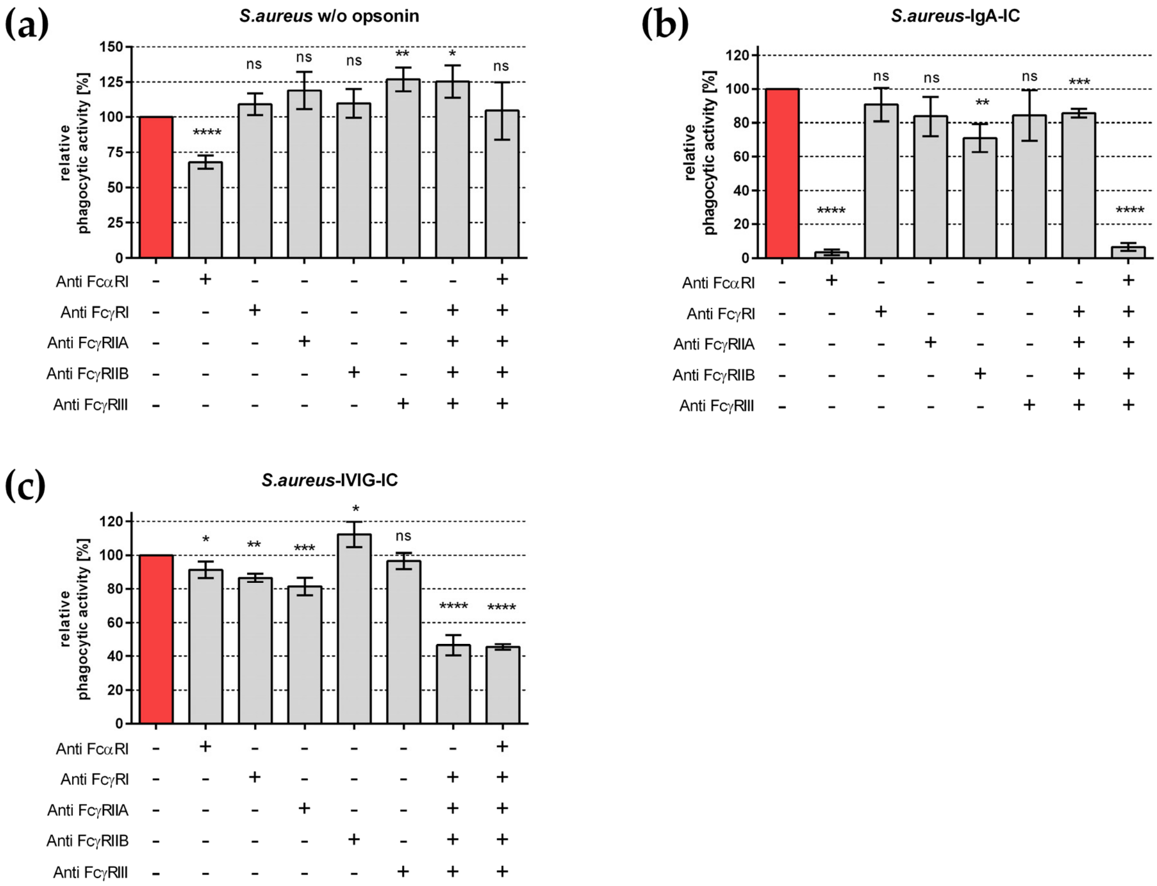

Figure S3. FcR blocking experiments (**a**) with *S.aureus* particles, (**b**) *S.aureus*-IgA-IC (**c**) and *S.aureus*-IVIg-IC. Neutrophil-like HL-60 cells were pre-incubated with 5 µg/mL of indicated blocking antibodies for 20 min. Phagocytic index of not blocked cells were referred to as 100 % phagocytic activity and the remaining phagocytic activity is shown. Values represent the mean of 6 independent experiments. Statistics: One way ANOVA; Dunnett's multiple comparisons test between not blocked cells and

indicated blocking. 95% confidence interval, * $p \leq 0.05$, ** $p \leq 0.01$, *** $p \leq 0.001$, **** $p \leq 0.0001$, ns = not significant.
